# Air Pollutant Concentration Forecasting Using Long Short-Term Memory Based on Wavelet Transform and Information Gain: A Case Study of Beijing

**DOI:** 10.1155/2020/8834699

**Published:** 2020-09-30

**Authors:** Bingchun Liu, Xiaoling Guo, Mingzhao Lai, Qingshan Wang

**Affiliations:** ^1^School of Management, Tianjin University of Technology, Tianjin 300384, China; ^2^School of Humanities, Tianjin Agricultural University, Tianjin 300384, China

## Abstract

Air pollutant concentration forecasting is an effective way which protects health of the public by the warning of the harmful air contaminants. In this study, a hybrid prediction model has been established by using information gain, wavelet decomposition transform technique, and LSTM neural network, and applied to the daily concentration prediction of atmospheric pollutants (PM_2.5_, PM_10_, SO_2_, NO_2_, O_3_, and CO) in Beijing. First, the collected raw data are selected by feature selection by information gain, and a set of factors having a strong correlation with the prediction is obtained. Then, the historical time series of the daily air pollutant concentration is decomposed into different frequencies by using a wavelet decomposition transform and recombined into a high-dimensional training data set. Finally, the LSTM prediction model is trained with high-dimensional data sets, and the parameters are adjusted by repeated tests to obtain the optimal prediction model. The data used in this study were derived from six air pollution concentration data in Beijing from 1/1/2014 to 31/12/2016, and the atmospheric pollutant concentration data of Beijing between 1/1/2017 and 31/12/2017 were used to test the predictive ability of the data set test model. The results show that the evaluation index MAPE of the model prediction is 7.45%. Therefore, the hybrid prediction model has a higher value of application for atmospheric pollutant concentration prediction, because this model has higher prediction accuracy and stability for future air pollutant concentration prediction.

## 1. Introduction

The rapid development of urbanization and industrialization has brought enormous economic results but also caused pressure on resources, energy, and the environment. The air pollution caused by the rapid development of urbanization and industrialization has become an important issue that restricts social and economic development and affects human health [1]. The six major atmospheric pollutants in the air (PM_2.5_, PM_10_, SO_2_, NO_2_, O_3_, and CO) are harmful to human health. Moreover, when the concentration of these pollutants exceeds the standard, it will destroy the human respiratory system and may cause a headache, dyspnea, and heart attack, which will seriously affect the health of human beings and thus restrict social development [2]. Therefore, it is vital to monitor and predict the concentration of pollutants, to avoid the health threats caused by excessive pollutant concentrations effectively. At the same time, the prediction of significant air pollution concentrations can be used as a policy tool for the environmental protection department to regulate social and economic activities such as transportation, industry, and urban construction under extreme air pollution conditions [3]. Therefore, in order to support the decision of environmental management and avoid serious accidents caused by air pollution, it is urgent to establish a precise and stable pollutant concentration prediction model, which can predict the concentration of air pollutants in the future, helping the government to publish control measures for air pollutants and public health protection work.

At present, the research on the prediction of atmospheric pollutant concentration in the world mainly focuses on the application of two methods: the deterministic model and the computational model. The deterministic model research does not require a large amount of historical data, but it needs to have complete knowledge of the source of pollution, the number of timely emissions, and the main chemical reactions and space-time physical transformation processes of the exhaust gases [4]. Computational models usually require a large amount of historical measurement data under various meteorological conditions, and the relationship between historical pollutant data and predicted variables is established by regression and neural network methods [5]. Sánchez et al. proposed a combination of three different methods, including Elman neural network, autoregressive integrated moving average, and a combination of these two methods, applied to predict a certain control near the coal-fired power plant station's SO_2_ concentration [6]. The results show that the hybrid method can still obtain excellent prediction results when the concentration of the particles is high. Wu et al. took Beijing as an example, using three-layer FFNN and regression Elman network, and the air quality prediction model was designed to predict the change of PM_10_ concentration of air pollutants on the next day [7]. The optimal model is then selected based on the test performance metrics and learning time. It is proved that the three-layer FFNN of the first-order line cut (OSS) training algorithm is superior to the Elman network of the gradient adaptive learning rate (GDX) training algorithm. As researchers continue to explore neural network algorithms, Das and Padhy constructed a new ANN model to predict PM_10_ concentrations in 23 EU countries [8]. This model reduces the average error of the results to less than 13% during the test. Subsequently, Elangasinghe et al. [9] extracted key information from daily available meteorological parameters and seasonal emission patterns and established a physics-based ANN air pollution prediction tool. The neural network model predicts better results than the linear regression model based on the same input parameters, and it can fully capture the temporal variation of air pollutant concentration in a specific scene. However, these models usually have a common defect; that is, the ability to predict the concentration of particulate matter such as PM_2.5_ and PM_10_ decreases at very high concentrations [5]. This flaw will mislead environmental control decisions and seriously affect human health. Therefore, more adequate experiments and sophisticated modeling techniques are needed to capture sudden changes in particle concentration. Taking this into account, Zhou et al. considered a low data dimension and used a hybrid EMMD-GRNN model based on data preprocessing and analysis to predict PM_2.5_ concentration one day ahead of time [10]. The model can be used to quickly and accurately predict the PM_2.5_ concentration for the next day. Wang et al. proposed an air pollutant prediction model based on a hybrid artificial neural network and hybrid support vector machine [11]. By modifying the error term of traditional methods, artificial neural network and support vector machine effectively improved the prediction accuracy. It has been noted that previous studies have often been used for single contaminant concentration predictions, ignoring the possible nonlinear correlations between different atmospheric contaminants [12]. Lv et al. established an empirical regression model for the prediction of PM_2.5_ and O_3_ air pollutant concentrations in three large Chinese cities in Beijing, Nanjing, and Guangzhou in 2016 [13]. The predictive model is an empirical nonlinear regression model designed for automated data retrieval and prediction platforms. The traditional neural network model cannot meet the requirements of high-precision, multioutput air quality prediction, and then the researchers improve the prediction accuracy by improving the input variable structure. Ni et al.'s results show that the selection of historical data such as PM_2.5_, PM_10_, temperature, wind direction, and wind speed on the previous day to train the model is crucial for the improvement of prediction accuracy [14]. On the other hand, Liu et al. proposed a new collaborative prediction model, using the SVR method of support vector regression to predict the Chinese urban air quality index AQI [15]. The experimental results show that when the air quality characteristic attribute and the air quality index when there are strong interaction and correlation between them, the MAPE value of the multicity multidimensional regression model is reduced.

Therefore, this study will use six major atmospheric pollutants as output variables, and the remaining five as input variables to explore the interactive prediction ability of various pollutants. Relative to the dynamic characteristics of the air pollution index, recurrent neural network (RNN) can effectively solve the adverse effects of the spatial and temporal evolution of the air pollution index. RNN is a deep learning method that can use any memory unit between networks to process any sequence in the input so that it can learn time series [16]. RNN technology has been proposed to solve the problem of time-series prediction, but studies have shown that the typical RNN model cannot solve the long-term dependence of the input sequence. In order to solve this problem, this paper uses a special long short-term memory artificial neural network (LSTM NN) of RNN structure. LSTM can learn time series of long spans and automatically determine the optimal time lag in the prediction. In recent years, LSTM has been successfully applied to image classification, natural language processing, human motion recognition, robot intelligence development, and oil price forecasting [17–19]. Therefore, based on the ability of LSTM to analyze and predict spatiotemporal data, this study applies it to the prediction of air pollution and can obtain good performance.

This research focuses on two aspects: (1) developing an LSTM atmospheric pollutant concentration prediction model based on deep learning; (2) optimizing the input indicators by selecting methods and data dimension processing to improve the prediction accuracy of the LSTM model. Taking Beijing as an example, the prediction of six major atmospheric pollutants (PM_2.5_, PM_10_, SO_2_, NO_2_, O_3_, and CO) was taken as the research object, and the stability and accuracy of the model were analyzed.

## 2. Methodology

### 2.1. Long Short-Term Memory

As the current popular recurrent neural network algorithm, the LSTM neural network was first proposed by Hochreiter and Schmidhuber, which improves the memory ability of long (static) and short (cyclic) dynamic features of time series [20]. Similar to the traditional cyclic neural network model, this model models the temporal data by mining the cyclical connections between neurons and mining the intrinsic connections between time-series data. However, unlike the traditional cyclic neural network model, it has a unique neuron structure called a “memory unit.” The hidden layer of the LSTM network constructed by the structure can store information of any length of time and obtain a more accurate time-series model [21].

**Figure 1:**
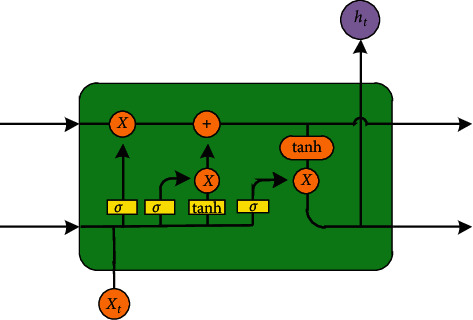
Schematic diagram of neurons.

The memory cell structure of the LSTM network is shown in Figure 1. The memory unit module is composed of three “door” structures of input gate, forgetting gate, and output gate and one loop unit. The core idea is to control the switching of each “gate” through a nonlinear function to protect and control the state of the memory unit, thereby controlling the increase and decrease of information [22]. Therefore, the key to the LSTM network is the long-term storage of data information through the state of the memory unit. In general, the three “gates” output value of 0-1 through the sigmoid function to determine how much information can be input to the memory unit.

Assuming that at time *t*, the input of a memory unit module is *x*_*t*_, the output is *h*_*t*_, and the unit state is *C*_*t*_, and then the formulas of input gate, forgetting gate, output gate, input conversion, unit state update, and output of the hidden layer of the memory unit module are shown in the following equations:

(1) $$\begin{array}{c} i_{t}=\sigma \left(W_{ix}x_{t}+W_{im}m_{t-1}+W_{ic}c_{t-1}+b_{i}\right), \end{array}$$

(2) $$\begin{array}{c} f_{t}=\sigma \left(W_{fx}x_{t}+W_{fm}m_{t-1}+W_{fc}c_{t-1}+b_{f}\right), \end{array}$$

(3) $$\begin{array}{c} o_{t}=\sigma \left(W_{ox}x_{t}+W_{om}m_{t-1}+W_{oc}c_{t}+b_{o}\right), \end{array}$$

(4) $$\begin{array}{c} C_{t}^{\prime }=\tanh\left(W_{cx}x_{t}+W_{cm}m_{t-1}+b_{c}\right), \end{array}$$

(5) $$\begin{array}{c} c_{t}=f_{t}⊗c_{t-1}+i_{t}⊗C_{t}^{\prime }, \end{array}$$

(6) $$\begin{array}{c} y_{t}=w_{ym}m_{t}+b_{y}. \end{array}$$

In this formula, *σ* is sigmoid function; tanh is hyperbolic tangent function; *i*_*t*_, *f*_*t*_, *o*_*t*_, and *C*_*t*_′ are input of input gate, forgetting gate, output gate, and input conversion pair unit, respectively; *W*_*ix*_, *W*_*fx*_, *W*_*ox*_, and *W*_*cx*_, and *W*_*im*_, *W*_*fm*_, *W*_*om*_, and *W*_*cm*_ are the weight matrix of the input gate, forgetting gate, output gate, and input conversion corresponding to *x*_*t*_ and *h*_*t*-1_, respectively; *b*_*i*_, *b*_*f*_, *b*_*o*_, and *b*_*c*_ are the offset vectors of input gate, forgetting gate, output gate, and input conversion, respectively [22].

### 2.2. Wavelet Transform

The wavelet transform is locally adjusted by the window adjustment, and the input signal is decomposed into a low-frequency signal capable of reflecting the true change trend of the signal data and a random disturbance high-frequency signal [23]. The contaminant concentration data are decomposed into a sequence group composed of different components by wavelet transformation. These subsequences have a more stable variance and fewer singular value points than the original data. Therefore, when using the LSTM model to predict the temporal data of atmospheric pollutants, in order to more accurately and accurately express the original signal information and improve the prediction accuracy, the input vector can be structurally transformed to increase the one-dimensional data to a higher level [24]. Air pollutant concentration time-series data {*y*_1_, *y*_2_,…, *y*_*n*_} can be considered as a set of signal sequences. Since wavelet analysis applies to such nonlinear and nonstationary time-series data, this method can be applied to analyze and extract the information characteristics of time-series data of atmospheric pollutant concentration at different frequencies [25]. The wavelet transform adjusts the window through the window to achieve the purpose of localized analysis and decomposes the input signal into a low-frequency signal that can reflect the actual change trend of the signal data and a random-disturbed high-frequency signal. The contaminant concentration data are decomposed into a sequence group composed of different components by wavelet transformation. Compared with the original data, these subsequences have more stable variance and fewer singular value points, which can express the original signal information more effectively and accurately, so the prediction accuracy is better. If the scaling function of the wavelet transform is *φ*(*t*), the parent wavelet function is *ψ*(*t*).

So,

(7) $$\begin{array}{c} \varphi_{j,k}\left(t\right)=2^{j/2}\varphi \left(2^{j}t-k\right), \end{array}$$

(8) $$\begin{array}{c} \psi_{j,k}\left(t\right)=2^{j/2}\psi \left(2^{j}t-k\right), \end{array}$$

where *j* and *k* are scale parameters and translation parameters respectively, and signal *y* (*t*) can be expressed by formulas (7) and (8) as follows:

(9) $$\begin{array}{c} y\left(t\right)=\sum_{k}c_{j_{o}}\left(k\right)2^{j_{0}/2}\psi \left(2^{j_{0}}t-k\right)+\sum_{k}\sum_{j=j_{0}}^{\infty }d_{j}\left(k\right)2^{j/2}\psi \left(2^{j}t-k\right), \end{array}$$

where *c*_*j*_0__(*k*) and *d*_*j*_(*k*) are estimated coefficients and detail coefficients, respectively, and the pollutant concentration data can be decomposed into *m* steps by wavelet transform:

(10) $$\begin{array}{c} y\left(t\right)=A_{mt}+D_{1t}+\cdots +D_{mt}. \end{array}$$

In formula (10), *a* is imperfect information representing the original information feature, and *b* is a piece of high-frequency information indicating a little signal fluctuation, that is, a noise portion of the original information. The low-frequency approximation information and the high-frequency information obtained by the wavelet transform decomposition constitute a new set of input vectors.

### 2.3. Information Gain

Usually, feature selection is always selected after quantifying the importance of the feature, and how to quantify the importance of the feature becomes the most significant difference between the various methods [26]. The correlation between features and categories is used in the square test to quantify. The stronger the association, the higher the feature score and the feature is more likely to be retained. In information gain, the measure of importance is to see how much information a feature can bring to a classification system [27]. The more information it brings, the more critical it is.

There is a variable *X*, which has more than *n* kinds of values, which are *x*_1_, *x*_2_,…, *x*_*n*_, respectively. The probability of each is *P*_1_, *P*_2_,…, *P*_*n*_, and then the entropy of *X* is defined as

(11) $$\begin{array}{c} H\left(X\right)=-\sum_{i=1}^{n}P_{i}\cdot \;\log_{2}P_{i}. \end{array}$$

For the classification system, category *C* is a variable, and its possible values are *C*_1_, *C*_2_,…, *C*_*n*_ and the probability of occurrence of each category is *P*(*C*_1_), *P*(*C*_2_),…, *P*(*C*_*n*_), So, *n* is the total number of categories. At this point, the entropy of the system can be expressed as

(12) $$\begin{array}{c} H\left(C\right)=-\sum_{i=1}^{n}P\left(C_{i}\right)\cdot \;\log_{2}\;P\left(C_{i}\right). \end{array}$$

In order to distinguish the symbol of *t* from the symbol of feature *t* itself, this paper uses *T* to represent the feature; then,

(13) $$\begin{array}{c} H\left(\left.C\right|T\right)=P\left(t\right)H\left(\left.C\right|t\right)+P\left(\bar{t}\right)H\left(\left.C\right|\bar{t}\right),\\ H\left(\left.C\right|t\right)=-\sum_{i=1}^{n}P\left(\left.C_{i}\right|t\right)\cdot \;\log_{2}\;P\left(\left.C_{i}\right|t\right). \end{array}$$

The other side can be expanded to

(14) $$\begin{array}{c} H\left(\left.C\right|\bar{t}\right)=-\sum_{i=1}^{n}P\left(\left.C_{i}\right|\bar{t}\right)\cdot \;\log_{2}\;P\left(\left.C_{i}\right|\bar{t}\right). \end{array}$$

Therefore, the information gain that the feature *T* brings to the system can be written as the difference between the original entropy of the system and the conditional entropy after the fixed feature *T*:

(15) $$\begin{array}{c} IG\left(T\right)=H\left(C\right)-H\left(\left.C\right|T\right)\\ =-\sum_{i=1}^{n}P\left(C_{i}\right)\cdot \;\log_{2}\;P\left(C_{i}\right)+P\left(t\right)\sum_{i=1}^{n}P\left(\left.C_{i}\right|t\right)\cdot \;\log_{2}\;P\left(\left.C_{i}\right|t\right)\\ +P\left(\bar{t}\right)\sum_{i=1}^{n}P\left(\left.C_{i}\right|\bar{t}\right)\cdot \;\log_{2}\;P\left(\left.C_{i}\right|\bar{t}\right). \end{array}$$

### 2.4. LSTM Forecasting Model

**Figure 2:**
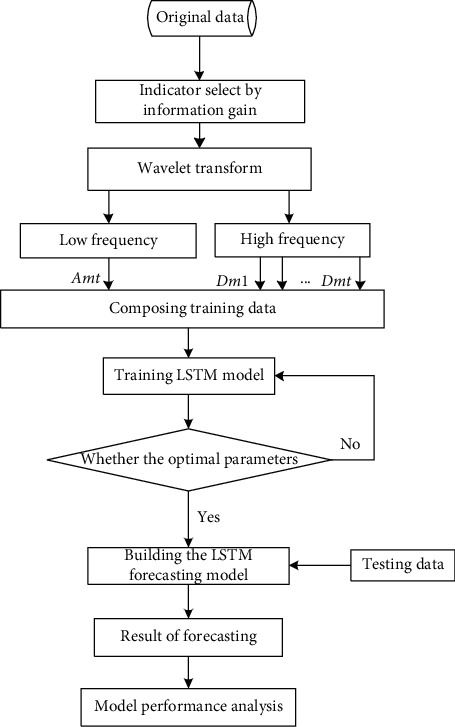
LSTM forecasting model.

The flow of the atmospheric pollutant concentration prediction model based on LSTM is shown in the following figure. The input variable consists of three parts, including the eigenvector group obtained by information gain, the high-frequency and low-frequency information vector group, and the historical data group, after wavelet decomposition transformation. In the design of the network structure, after repeated experimental debugging, the complete LSTM model was finally determined, as shown in Figure 2. The entire LSTM neural network contains N LSTM hidden layers, and each layer contains 256 nodes.

The detailed steps for running the model are as follows:

- Step 1: form the time-series data set {*AP*_1_, *AP*_2_,…, *AP*_*n*_} from the air pollutant concentration data, use information gain to select the input index characteristics of different prediction targets in the air pollutant concentration prediction, respectively, and obtain the significant factor data set {*I*_1_′, *I*_2_′,…, *I*_*t*_′}.
- Step 2: using the wavelet decomposition transform, the significant factor data set {*I*_1_′, *I*_2_′,…, *I*_*t*_′} is decomposed into *m* layers to obtain a high-dimensional input information set {*X*_1_′, *X*_2_′,…, *X*_*t*_′}, where *X*_*i*_′=(*A*_*mi*−1_, *D*_1*i*−1_,…, *D*_*mi*−1_), *i*=1,…, *t*, and *t*+1 decompose the result *X*_*t*+1_′=(*A*_*mt*_, *D*_1*t*_,…, *D*_*mt*_) at the moment.
- Step 3: construct a new training data set {(*X*_*i*_′*Y*_*i*_′)}_*i*=1_^*t*^ based on the high-dimensional input information {*X*_1_′, *X*_2_′,…, *X*_*t*_′} obtained in the second step.
- Step 4: use the new group dataset {(*X*_*i*_′*Y*_*i*_′)}_*i*=1_^*t*^ to train the LSTM model, and through trial and error, adjust the parameters to get the prediction mode *f*(*X*_*i*_).
- Step 5: using the prediction model obtained from the above training and the *t*+1 stage input vector *X*_*t*+1_ obtained in Step 1, the predicted value *f*(*X*_*t*+1_′) of the *t*+1 stage atmospheric pollutant concentration can be measured.

The predicted value *f*(*X*_1_′),…, *f*(*X*_*t*+1_′) will be obtained by repeating Step 1–Step 5.

## 3. Research Object and Exploratory Data Analysis

The object of this study is Beijing, which is located in the northern part of the North China Plain. It consists of 16 functional areas in 6 districts and 10 suburbs, with a total area of 16,410.51 km^2^. Beijing belongs to 39.4°N to 41.6°N and 115.7°E. To 117.4°E.

Collect historical data on the concentration of atmospheric pollutants in Beijing from January 1, 2013, to December 31, 2017. The ground measures six major pollutant concentrations per hour (PM_10_, PM_2.5_, NO_2_, SO_2_, O_3_, and CO). The data collected are from the China National Environmental Monitoring Centre (http://www.cnemc.cn/) and the Ministry of Ecology and Environment of the People's Republic of China (http://www.zhb.gov.cn/). The statistical description of the main indicators is shown in Table 1.

**Figure 3:**
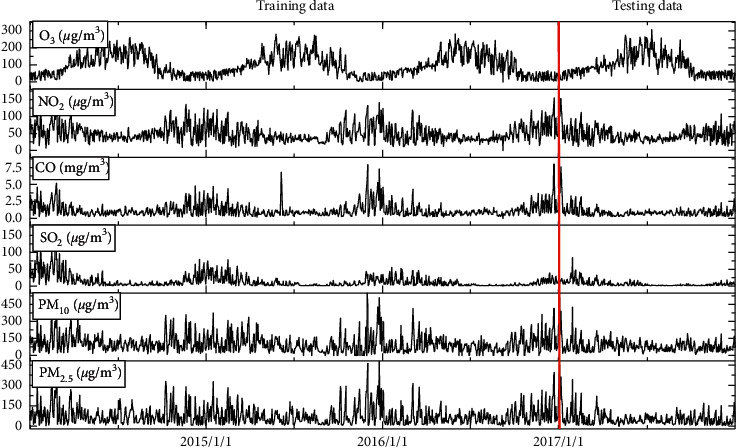
Original data (2014/1/1–2017/12/31).

When using the deep learning method to build the model, the selection and normalization of features are essential for the performance of the model. Through exploratory analysis, it is found that there are apparent abnormal fluctuations in the raw data of pollutant concentration with seasonal changes, as shown in Figure 3. Due to the Beijing Huilongguan fire accident on June 5, 2015, the CO concentration suddenly increased to 6.8 *μ*g/m^3^, and such abnormal fluctuations will seriously affect the predictive ability of the model. Therefore, this study introduces the wavelet decomposition transform; the original data are transformed to obtain low-frequency data and high-frequency data subsequences. These subsequences have more stable variance and fewer singular value points than the original data, which makes the input vector smooth. The concentration of O_3_ has increased significantly in the same month in the same period of four years and has become the main factor of air pollution in Beijing. Under the influence of environmental management policies such as Beijing's industrial migration, the concentrations of PM_10_ and PM_2.5_ have decreased year by year. However, due to the increasing number of private cars in Beijing, the concentration of nitrogen oxides has increased significantly, which has become a new major air pollution factor.

In order to study the mutual prediction ability between the six major atmospheric pollutants in Beijing, this study will use a total of 1095 data from January 1, 2014, to December 31, 2016, as the training data set for the prediction model. Others include 2017. Three hundred sixty-five data from January 1 to December 31, 2017, were used as test data sets.

## 4. Results and Discussion

### 4.1. Result of Information Gain

This study uses nonpredictive targets as input vectors. The purpose is to cope with the specific pollution indicators that can be obtained in the prediction environment with a strong influence of complex, uncertain factors, use the clear indicators to predict each other, and obtain the prediction model with higher applicability. In order to improve the accuracy of model prediction, this study will further select the input variables, select the input variables by information gain, determine the correlation degree with each pollutant concentration, and screen the significant indicators. Using the six atmospheric pollutants (PM_10_, PM_2.5_, NO_2_, SO_2_, O_3_, and CO) in the original data set as targets, respectively, through information gain to complete correlation exploration and feature selection, we can obtain the entropy of each input variable for the prediction target. The value is sorted as shown in Table 2.

Table 3 lists the three items that are most important in predicting pollutants in order. It can be known from the table that PM_10_ and PM_2.5_ have a dangerous influence on the concentration prediction of SO_2_, NO_2_, O_3_, and CO when they are used as input variables, and there are the two pollutants with the most substantial influence on the results during the prediction of atmospheric pollutant concentration. It may be because its source has a specific correlation, which is derived from the burning of fossil fuels such as automobile exhaust. The research model does not consider the complex and uncertain prediction environment. Therefore, in order to improve the accuracy of the prediction model, only the feature vector acquired by the IG is used as an input variable for model training.

### 4.2. Result of Wavelet Decomposition

Although LSTM is applied to time-series prediction, it can show good prediction performance. However, the LSTM model efficiently represents the high-dimensional nonlinear relationship between the input vectors and the predicted targets through the kernel function. The appropriate high-dimensional input vectors can be used to describe the information features more effectively and accurately and express the meaning of the data. Therefore, model prediction ability depends on no small extent on the choice of input vectors in the model design. In this study, when using the LSTM model to predict the concentration of pollutants, in order to make the prediction results more accurate and stable, the input variables can be structurally transformed to obtain a new set of input variables. The data are upgraded from one-dimensional to high-dimensional data by wavelet decomposition, which more fully and adequately represents the trend of data changes, thereby improving the prediction accuracy. In this study, wavelet decomposition is based on Daubechies (DB) wavelet basis function [4]. Daubechies has low-pass and high-pass filtering properties, which is suitable for feature selection. Because of its inherent orthogonality, Daubechies wavelet can be widely used and shows good performance in time-series data of analysis applications.

**Figure 4:**
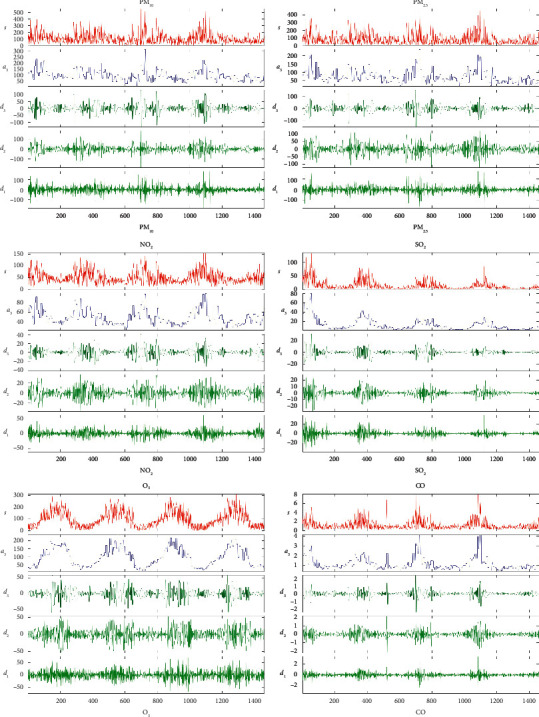
The result of wavelet transform.

Matlab tool was used to form a new prediction data set of six atmospheric pollutants (PM_10_, PM_2.5_, NO_2_, SO_2_, O_3_, and CO) using the low-frequency approximate information and high-frequency information obtained by wavelet decomposition transformation, respectively, as another new input vector group of the LSTM model. The result of the transformation is shown in Figure 4, which is a high-frequency information group and is a low-frequency information group. The wavelet decomposition set generates high-dimensional input vectors by wavelet decomposition transformation from the density time-series data of three input feature variables, which can effectively increase the data representing information, and the prediction stability of the prediction model is significantly improved.

### 4.3. Determination of the Best Parameters of LSTM Model

In order to ensure that the hybrid model obtains the best experimental results, the best parameters of the LSTM model should be determined before the experiment starts, so as to reduce the influence of parameter factors on the experimental results. There are three major parameters in the LSTM model, namely, the number of time steps *L* of each layer in the LSTM, the size of the hidden unit (the same hidden unit is used for each layer in the LSTM), and the batch size during training. Also it includes learning rate (Lr) and max epochs. When selecting the experiment for predicting the best model in the experiment, the number of frames in each sample is set to *L*. When one parameter is different, the other parameters are fixed, and finally, the best prediction model is found. The model parameters are shown in Table 4.

### 4.4. Result of Hybrid Model

In order to investigate the performance of the LSTM atmospheric pollutant concentration prediction model, this study provided four sets of input variables for the training prediction model, namely, Beijing atmospheric pollutant concentration original data set, characteristic variable set, high-dimensional data set, and high-dimensional characteristic variable set. Verifying the high prediction accuracy of the hybrid LSTM model established in this study, six predictions of atmospheric pollution concentration using different methods for feature selection were compared in this study. This prediction model in the experiment was designed by Matlab 2017a and Ubuntu system using Python 2.7 programming. The minimum MAPE was selected as the target for the selection of relevant parameters in the model. Mean absolute percentage error (MAPE) is an important indicator to measure the accuracy of prediction in the statistical field [28].

In this paper, the MAPE index is also used to measure the error of the load prediction algorithm and compare it with other algorithms. MAPE not only considers the error between the predicted value and the real value but also considers the proportion between the error and the real value. The following formula gives the calculation expression of MAPE:

(16) $$\begin{array}{c} e^{\text{MAPE}}=\frac{100\%}{N}\sum_{t=1}^{N}\left|\frac{L_{t}^{a}-L_{t}^{f}}{L_{t}^{a}}\right|, \end{array}$$

where *e*^MAPE^ represents the load prediction error measured by the MAPE index; *N* represents the total number of load prediction time points; *L*_*t*_^*a*^ represents the actual load value; and *L*_*t*_^*f*^ represents the predicted load.

At the beginning of the experiment, the LSTM model with four hidden layers was selected. The original data of six atmospheric pollutants were used as the prediction targets, and the remaining five were used as independent variables. When PM_10_ is used as the dependent variable, MAPE can be as low as 7.54%, and when PM_2.5_ is used as the dependent variable, MAPE reaches 17.25%. The predicted results of the MAPE standard deviation are substantial. The above results show that the prediction stability of the model cannot be guaranteed unless when the independent variables are different using the LSTM model. Therefore, in order to improve the stability and accuracy of the LSTM prediction, the learning efficiency of the auxiliary vector enhancement model is added to the prediction model. Through the feature selection of information gain, only the feature variable set consisting of three input variables with higher correlation is used to train the LSTM model. The average MAPE of the prediction result is reduced from 12.62% to 10.75%, and the prediction accuracy of the model is improved. However, the CO prediction result is improved. The MAPE is 4.35%, and the PM_2.5_ prediction result is 17.85%. The stability of the prediction model is still not guaranteed. In order to achieve the effect of improving the data dimension, the three sets of high-frequency information and a set of low-frequency information obtained by wavelet decomposition are used to form a high-dimensional data set. The subsequence data transformed by the wavelet decomposition have a more stable variance and fewer singular value points than the original data and can express the original signal information more effectively and accurately. After the LSTM model was fitted using only G2 as training data, the average MAPE was 10.65%, which was 0.05% lower than the G2-assisted prediction alone. However, the variance of the model for different predicted target MAPE is significantly smaller, and the stability can be significantly improved. Therefore, it can be concluded that the time-series data after wavelet decomposition has more stable prediction performance in prediction.

**Figure 5:**
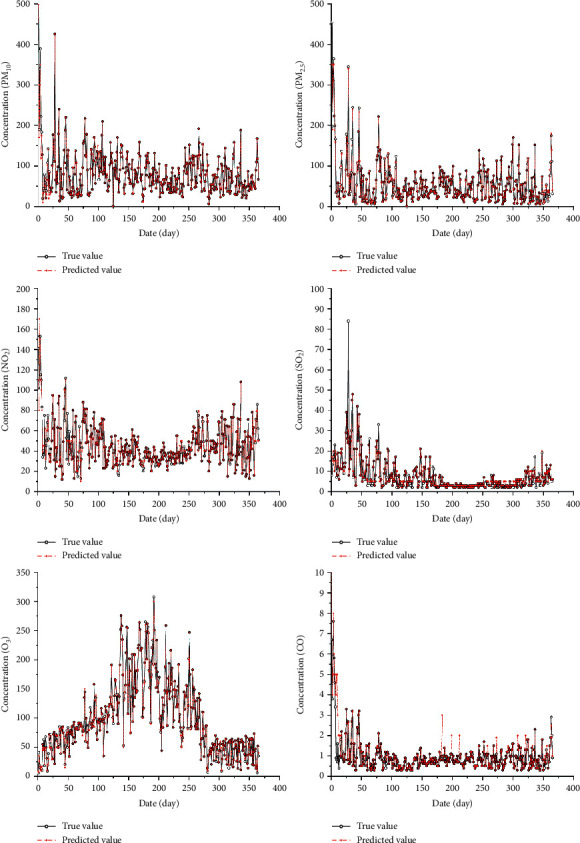
The concentration forecasting results of the hybrid LSTM model.

**Figure 6:**
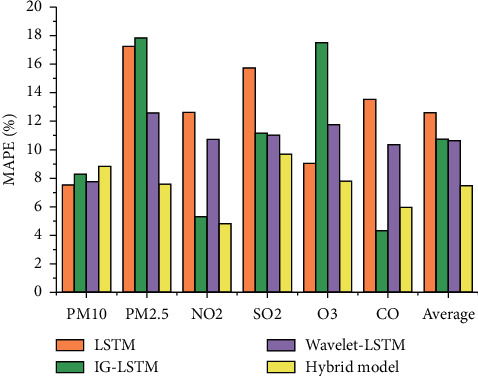
MAPE of models.

On this basis, this study attempts to set up a new training dataset after the original data of atmospheric pollutants are selected by information gain features and dimensionality enhanced by wavelet transform, aiming at smoothing the training data set of the deep learning model and enhancing the learning ability of the LSTM model. The average MAPE of the prediction result is reduced to 7.45% when the LSTM model is trained with the high-dimensional characteristic data set.  Figure 5 shows that when the input vector is processed using wavelet transform and IG, the prediction accuracy is higher and the stability is excellent. The evaluation of the prediction results of each type of air pollution prediction under different models is shown in Table 5 and Figure 6. The actual and predicted concentrations of various atmospheric pollutants in 2017 are compared as shown in Figure 5.

LSTM has achieved excellent results as a high-dimensional nonlinear learning algorithm for the prediction of atmospheric pollutant concentration's time-series data. However, due to the incomplete representation of one-dimensional time-series data, the generalization ability of the prediction model is restricted to some extent. The hybrid prediction model proposed in this study uses wavelet to decompose the time-series data of various pollutant concentrations, constructs new high-dimensional feature vectors to express the relevant information of different pollutants at different frequencies, and better displays the data characteristics.

In order to evaluate the forecasting performance of the hybrid model more comprehensively, the experiment compares the model with some state-of-the-art time-series prediction models, including machine learning methods and deep learning methods. In the experimentation, the SVR, RNN, and GRU models were selected as the comparison models. Furthermore, all the control models were combined with the IG and wavelet model. The training set and the test set were the same as for the hybrid model. A total of three groups of comparative experiments were performed. The final results are shown in Table 6. From the results, It can be seen that the prediction model proposed in this paper improves the accuracy of prediction and is more effective than other prediction models.

### 4.5. The Stability of the Hybrid Models

In order to measure and evaluate the stability of the hybrid models, the experiment collected data from different regions for verification, including Tianjin (TJ), Shanghai (SH), and Shijiazhuang (SJZ). The characteristics of the data collected from different regions are also different. Tianjin is also located in the northern part of the North China Plain, and the level of air pollution is similar to Beijing (BJ). Shanghai is situated in the Yangtze River delta, and the level of air pollution is milder to Beijing. Shijiazhuang is located in the middle of the North China Plain, and the level of air pollution is more serious than Beijing. The data of three regions are reforecasted to evaluate the stability of the hybrid models. The forecasting procedure and model parameters of three regions is the same as that in Beijing which removes the need to make some duplicate figures. The final prediction results are shown in Table 7.

As shown in Table 7, the MAPE evaluation indicators of the three regions are relatively reasonable, and the average values are close to those of Beijing. Especially, in Tianjin, the average value is 7.21%, which is the closest to Beijing's 7.45%, and the level of air pollution is similar to Beijing. This shows that the hybrid model has high stability and is suitable for air pollution concentration prediction.

## 5. Conclusions

Severe air pollution has a significant impact on human health, flora and fauna, and the environment. For human health, dangerous air pollution is prone to respiratory diseases and physiological dysfunction, which severely hinders human health. Therefore, scientific and accurate prediction of the concentration of atmospheric pollutants has an important practical significance, which can provide current prediction data and basis for environmental protection agencies, reduce the impact of air pollution on people's health, and guide people's work and life. In this paper, a hybrid LSTM model is established based on wavelet decomposition, information gain (IG), and long- and short-term neural network (LSTM) methods to predict the concentration of six major atmospheric pollutants in Beijing in the future. The study is summarized as follows:

1. Using the information gain method to select the input variables is helpful to improve the accuracy of the LSTM neural network prediction results of six air pollution concentrations and use wavelet decomposition to convert the characteristic input variables into high-dimensional data. The collection effectively enhances the stability and accuracy of the predictive model.
2. The hybrid prediction model uses wavelet decomposition of atmospheric pollutant concentration's time-series data, and the low-frequency data and high-frequency data obtained after decomposition are simultaneously used as input variables, increasing the data dimension. The information performance of the pollutant concentration's time-series data at different frequencies is better described.
3. It can be seen from the experimental results that the hybrid prediction model has a significant improvement in the prediction accuracy of pollutant concentration and increased instability. Especially, in the prediction of burst data points, the hybrid prediction model can predict more accurately. Therefore, the high-dimensional input variable pair's data information composed of low-frequency information and high-frequency information obtained by wavelet transform can be considered as the pollutant concentration's time-series data make this expression more accurate.
4. This study uses historical data to obtain the calculation results of the characteristic entropy of other pollutant concentrations when different pollutants are used as predictive target variables. According to the experimental results, it can be observed that the characteristic entropy of the concentration of PM_10_ and PM_2.5_ for the concentration prediction of most major atmospheric pollutants is significant, indicating that when insufficient fossil fuels lead to the increase of SO_2_ and NO_2_ and other pollutants, the concentration of PM_10_ and PM_2.5_ in the air will be severely affected.
5. The LSTM neural network method can be used in the model to obtain the prediction results of atmospheric pollutant concentration more accurately. This prediction model is applied to the prediction of six air pollutant concentrations in Beijing. By comparing the actual data, the average MAPE predicted is as low as 7.45%. Compared with the mechanism model with complex and high computational cost, it is more suitable for the prediction environment with robust and complex uncertainty factors. Therefore, the hybrid prediction model has strong applicability and high application value in predicting the concentration of atmospheric pollutants.
6. By predicting the concentration of air pollutants in three different regions, it can be seen that the hybrid model is more stable and the forecast of air pollution concentration is more reliable. In the control experiment, the MAPE of the other three regions is close to the value of Beijing, indicating that the hybrid model can still obtain better prediction results under the condition of different characteristic data values, and the model has good stable prediction performance.

## Figures and Tables

**Table 1:** Statistical descriptions of the main indicators.

	PM_2.5_	PM_10_	NO_2_	CO	SO_2_	O_3_
Unit	*μ*g/m^3^	*μ*g/m^3^	*μ*g/m^3^	*μ*g/m^3^	*μ*g/m^3^	*μ*g/m^3^
Mean	73.63	99.24	12.76	1.17	49.45	98.49
Std.	65.73	73.89	15.84	0.96	23.68	64.68
Min	5	7	2	0.2	10	3
Max	477	550	133	8	155	308
Median	55	84	7	0.9	43.5	84.5
Skewness	2.02	1.79	2.91	2.83	1.19	0.68
Kurtosis	5.49	4.79	11.21	11.52	1.56	−0.35

**Table 2:** Feature selection results.

	PM_10_	PM_2.5_	NO_2_	SO_2_	O_3_	CO
PM_10_	*∗*	3.4629	2.7605	1.3870	3.6033	1.8323
PM_2.5_	1.8323	*∗*	2.7416	2.7416	3.4725	1.8708
NO_2_	3.6033	1.8708	*∗*	2.7604	1.9318	1.3870
SO_2_	3.4629	3.4725	2.6294	*∗*	1.7792	0.9957
O_3_	2.0010	1.9008	1.4521	1.4521	*∗*	1.7791
CO	2.7416	2.6294	1.3870	2.6294	2.7605	*∗*

**Table 3:** The results of feature selection.

	PM_10_	PM_2.5_	NO_2_	SO_2_	O_3_	CO
1	NO_2_	SO_2_	PM_2.5_	PM_2.5_	PM_10_	PM_2.5_
2	SO_2_	PM_10_	PM_10_	NO_2_	PM_2.5_	PM_10_
3	CO	CO	SO_2_	CO	CO	O_3_

**Table 4:** Parameters for the LSTM network.

Parameter *t*	Time_steps	Hidden_layers	Batch_size	Lr	Epoch
LSTM	2	64	2	0.001	5000

**Table 5:** MAPE of models (%).

	PM_10_	PM_2.5_	NO_2_	SO_2_	O_3_	CO	Average
LSTM	**7.54**	17.25	12.63	15.73	9.05	13.53	12.62
IG-LSTM	8.30	17.85	5.31	11.17	17.51	**4.35**	10.75
Wavelet-LSTM	7.78	12.59	10.73	11.02	11.77	10.37	10.71
Hybrid model	8.84	**7.59**	**4.83**	**9.69**	**7.80**	5.96	**7.45**

**Table 6:** Comparison of prediction accuracy of different models (%).

	PM_10_	PM_2.5_	NO_2_	SO_2_	O_3_	CO
Hybrid model	**8.84**	**7.59**	**4.83**	**9.69**	**7.80**	**5.96**
IG-wavelet-SVR	16.39	17.23	21.29	22.36	20.75	21.55
IG-wavelet-RNN	12.60	12.28	13.51	16.96	12.21	9.48
IG-wavelet-GRU	10.09	9.92	13.50	10.10	9.78	7.75

**Table 7:** Stability measure and evaluation of the hybrid model (%).

	PM_10_	PM_2.5_	NO_2_	SO_2_	O_3_	CO	Average
Hybrid model (TJ)	10.07	7.09	6.74	4.22	6.85	8.34	7.21
Hybrid model (SH)	3.21	6.73	5.46	6.72	4.98	11.63	6.45
Hybrid model (SJZ)	10.26	9.35	13.21	7.61	8.75	11.68	10.14
Hybrid model (BJ)	**8.84**	**7.59**	**4.83**	**9.69**	**7.80**	**5.96**	**7.45**

## Data Availability

Raw data used to support the results of this study are included in the article.
